# Correction: Emerging opportunities in the rewiring of biology through proximity inducing small molecules

**DOI:** 10.1039/d5md90040a

**Published:** 2025-10-07

**Authors:** Michael M. Hann

**Affiliations:** a Independent consultant (formerly senior research director GSK) Letchworth UK mikehann54@btinternet.com

## Abstract

Correction for ‘Emerging opportunities in the rewiring of biology through proximity inducing small molecules’ by Michael M. Hann, *RSC Med. Chem.*, 2025, https://doi.org/10.1039/d5md00608b.

The author regrets that an incorrect enzyme, CDK8, is stated within the text in the antepenultimate paragraph of the article. The corrected version of the affected text is “What is particularly novel about this example is that the part of the bifunctional molecule that recruits the CDK9 enzyme to phosphorylate BCL6 is actually an inhibitor of CDK9! The kinetics of this system must be finely balanced such that the inhibitory ligand for CDK9 can help relocate it to the vicinity of the BCL6 and then release it to carry out the required phosphorylation.”

The Royal Society of Chemistry apologises for these errors and any consequent inconvenience to authors and readers.

